# Non-exposed endoscopic wall-inversion surgery as a novel partial gastrectomy technique

**DOI:** 10.1007/s10120-013-0291-5

**Published:** 2013-08-23

**Authors:** Takashi Mitsui, Keiko Niimi, Hiroharu Yamashita, Osamu Goto, Susumu Aikou, Fumihiko Hatao, Ikuo Wada, Nobuyuki Shimizu, Mitsuhiro Fujishiro, Kazuhiko Koike, Yasuyuki Seto

**Affiliations:** 1 Department of Gastrointestinal Surgery, Graduate School of Medicine, The University of Tokyo, 7-3-1, Hongo, Bunkyo-ku, Tokyo, 113-8655 Japan; 2 Department of Endoscopy and Endoscopic Surgery, Graduate School of Medicine, The University of Tokyo, Tokyo, Japan; 3 Department of Gastroenterology, Graduate School of Medicine, The University of Tokyo, Tokyo, Japan

**Keywords:** Full-thickness resection, Gastrointestinal stromal tumor, Partial gastrectomy, Submucosal tumor, Laparoscopic gastrectomy

## Abstract

In gastric full-thickness resection employing both endoscopy and laparoscopy, intraabdominal contamination or even possibly tumor seeding is unavoidable as a result of iatrogenic perforation and the resultant spread of gastric juice. To minimize contamination and resected tissue volume, we developed a new technique without perforation termed non-exposed endoscopic wall-inversion surgery (NEWS), and present here the preliminary results. In a clinical observation cohort study, NEWS was attempted in six patients with gastric SMT to investigate the procedure, mortality, and morbidity. NEWS consists of several steps: marking around a tumor on the mucosal as well as the serosal surface, submucosal injection of sodium hyaluronate with indigo carmine dye, circumferential seromuscular dissection with suture closure under laparoscopy, and circumferential mucosubmucosal incision under gastric endoscopy. The resected specimen is then retrieved perorally. Perforation occurred as a result of misidentification and technical inadequacy in the first three patients. After modification of the devices, the entire procedure was successfully achieved in the latter three. There were no complications in any of our six cases. NEWS allows en bloc full-thickness resection, theoretically avoiding contamination and tumor dissemination into the peritoneal cavity.

## Introduction

Laparoscopic wedge resection has been widely accepted and is now considered to be a minimally invasive surgery for small gastrointestinal stromal tumor (GIST) in the stomach [1]. Simple resection using a linear stapler is technically easy, although unnecessary excessive resection of unaffected gastric wall is generally unavoidable.

Laparoscopic and endoscopic cooperative surgery (LECS) [2, 3] and laparoscopic intragastric surgery (LIGS) [4] have been advocated in efforts to minimize the area to be resected. In fact, these procedures minimize the surgical specimen and provide better outcomes [5, 6]. However, these methods also carry inherent risks of peritoneal infection because of the necessity of gastric perforation. Although the exact incidence of peritoneal contamination with these procedures has yet to be determined, it has become evident that iatrogenic gastrotomy leads to seeding of bacteria in animal models [7, 8] and in humans as well [9]. Although gastrotomy and the resultant bacterial spillage are not associated with severe septic complications [9, 10], avoidance of contamination is undoubtedly preferable.

We previously demonstrated that a new technique of gastric full-thickness resection is technically feasible and safe, in an ex vivo model [11] and an in vivo survival model [12]. This procedure, at least theoretically, would minimize the resected tissue volume as well as prevent peritoneal contamination. We herein report a small series of patients with suspected gastric GIST treated by this new technique, termed non-exposed endoscopic wall-inversion surgery (NEWS).

## Patients and methods

### Patients

Between July 2011 and September 2012, we performed NEWS on six patients with suspected small gastric GIST. Tumors of the exophytic growth type were excluded. The protocol was approved by the institutional ethics review board of our university, and informed consent was obtained from all the patients.

### Procedure

**Fig. 1:**
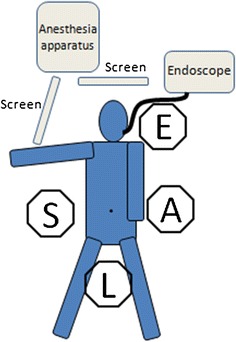
Position of the study participants in the operating room: *S* surgeon, *A* assistant, *L* laparoscopist, *E* endoscopist

The patient was placed in the supine position with the legs apart. A surgeon, a first assistant, a laparoscopist, and an endoscopic operator were positioned as shown in Fig. 1) One camera port was primarily inserted in the umbilical portion, and pneumoperitoneum was established. Then, 5-mm trocars were placed in the left upper, left lower, and right upper quadrants and a 12-mm trocar in the right lower quadrant, five trocars in total.

**Fig. 2:**
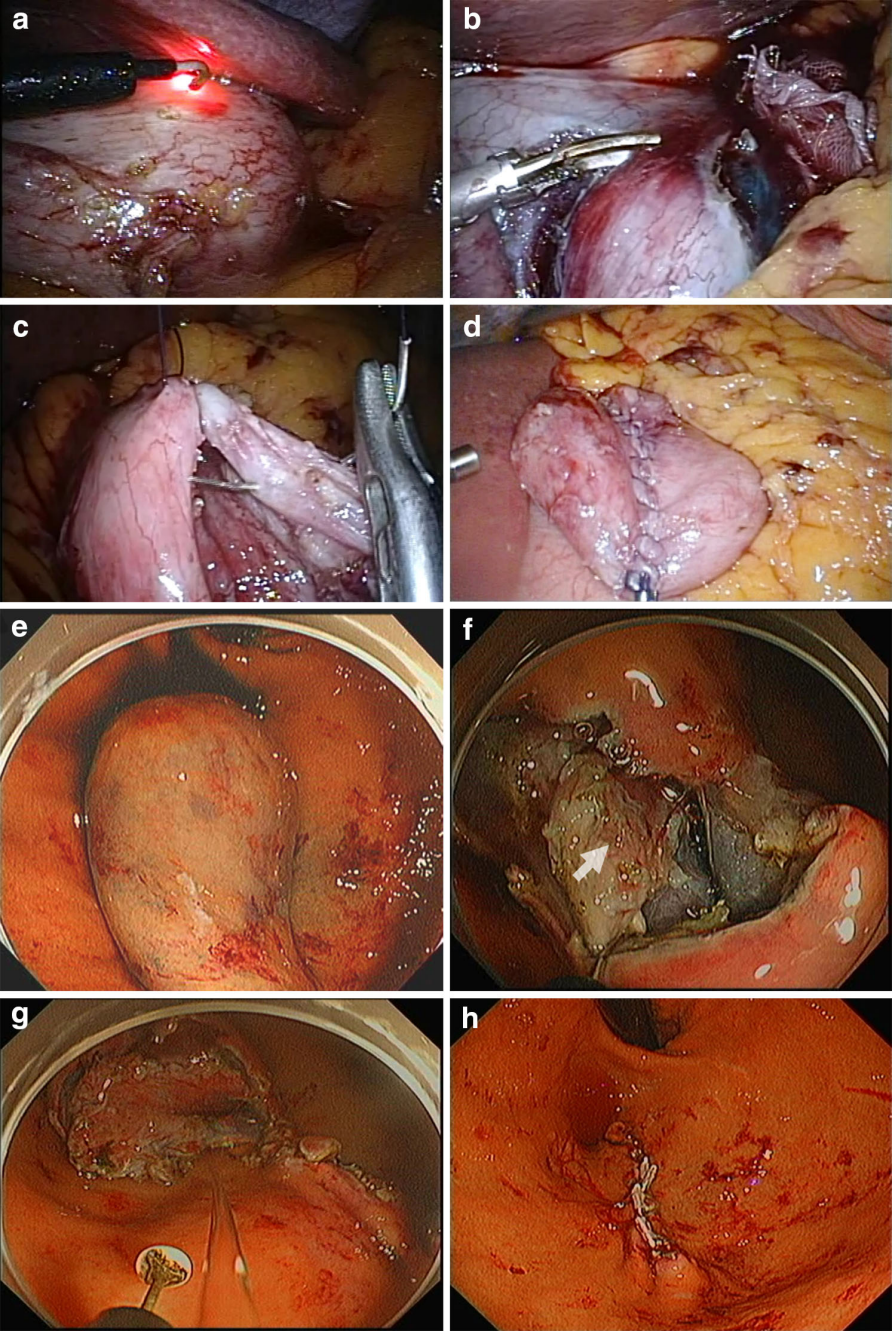
Procedures of non-exposed endoscopic wall-inversion surgery. **a** Laparoscopic markings on the serosal surface guided by light from the fiberoptic probe shining through the gastric endoscope. **b** Circumferential seromuscular dissection outside the serosal markings. **c**, **d** Seromuscular suture closure and spontaneous inversion of the dissected area. **e** Gastric endoscopic images. Massive protrusion of the inverted tissue. **f** Serosal surface (*arrow*) identified during mucosubmucosal dissection. **g** Flipped tissue to be resected. **h** *Dissected lines* of the mucosal surface were spontaneously combined and closed using clipping devices

The tumor location was confirmed employing a flexible endoscope with a carbon dioxide supplier. Markings were made on the mucosa around a lesion with the tip of a Dual knife (KD-650L; Olympus Medical Systems, Tokyo, Japan). Accordingly, serosal markings were made laparoscopically on the side opposite the mucosal markings with a hook knife, guided by pressing the gastric wall using the tip of a Flex knife, or the fiberoptic probe of a diode laser system (UDL-60; Olympus) (Fig. 2a). A 0.4 % sodium hyaluronate solution with a small amount of indigo carmine dye was endoscopically injected into the submucosal layer circumferentially.

**Fig. 3:**
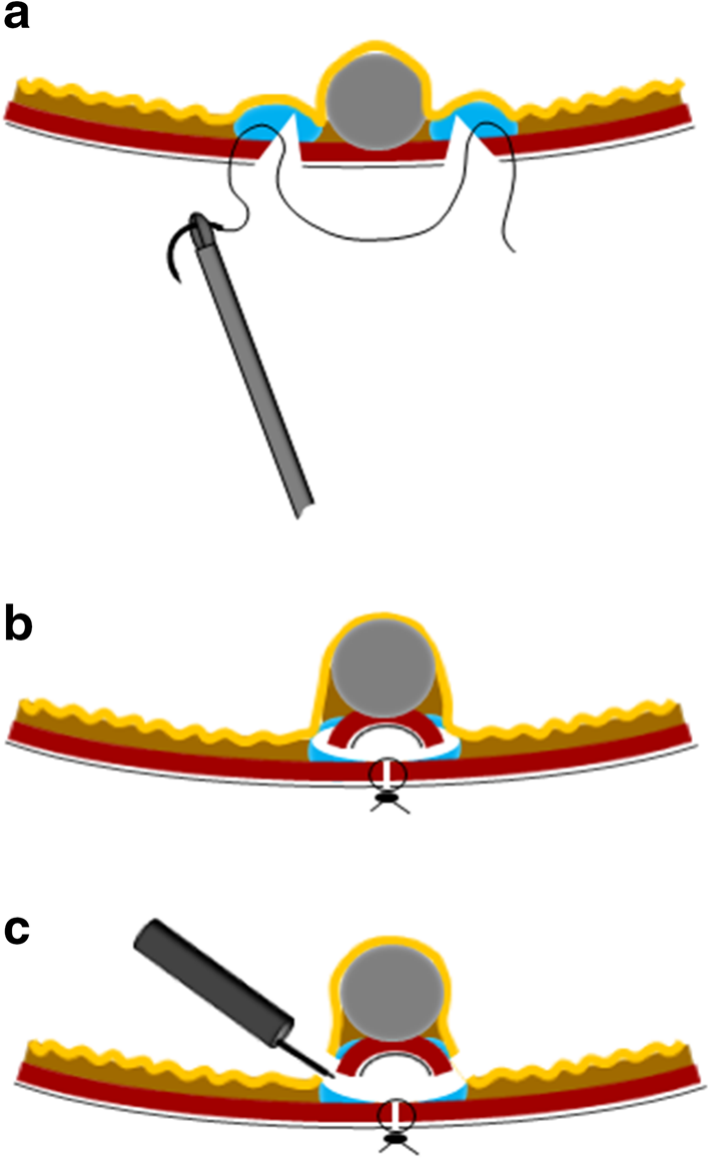
Scheme of the procedure. **a** Seromuscular layer suture after submucosal injection and seromuscular cutting. **b** Divided seromuscular layer inversion after laparoscopic seromuscular closure. **c** Mucosubmucosal layer is cut by the endoscopic device

A circumferential seromuscular incision was laparoscopically made around the serosal markings with the hook knife or an energy surgical device (Harmonic Ace; Ethicon Endo-Surgery) (Fig. 2b). The seromuscular layer was continuously sutured using 3-0 absorbable braided suture (Figs. 2c, 3a), allowing spontaneous inversion of the lesion (Fig. 2d, e). The mucosubmucosal layer was circumferentially incised outside the mucosal markings with a dual knife and an IT knife2 (KD-611L; Olympus) using endoscopic submucosal dissection (ESD) techniques (Fig. 2f, g). After the lesion removed, we closed the mucosal layer optionally by the endoscopic clipping device even when seromuscular anastomosis has been established [13, 14]; (Fig. 2h).

The specimen was extracted using an endoscopic retrieval device (Roth net retriever-polyp; US endoscopy, OH, USA).

## Results

**Fig. 4:**
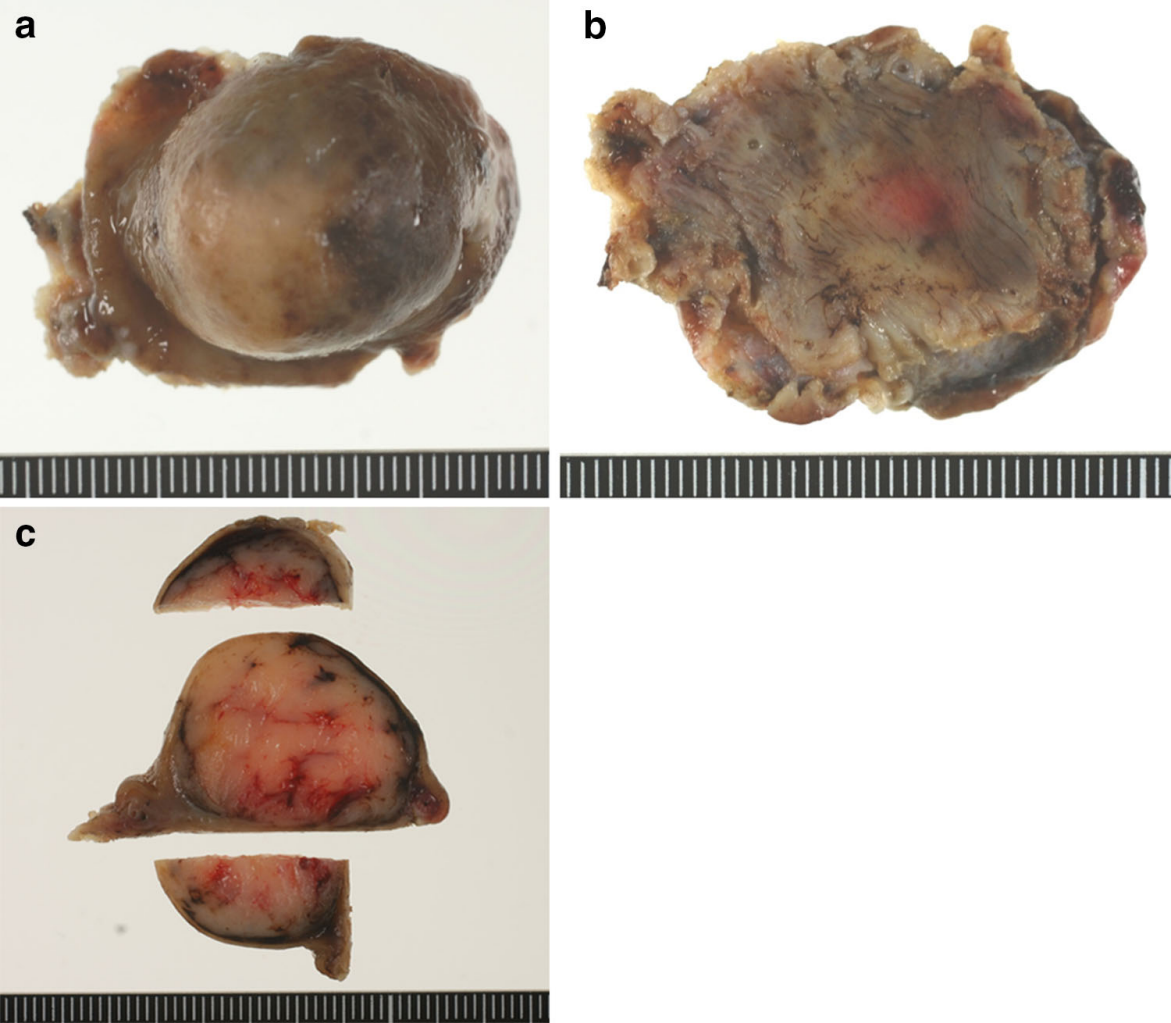
Representative resected tissue. **a** View from mucosal side. **b** Serosal side. **c** Cross section

The clinicopathological characteristics of our series are described in Table 1. Mean patient age was 63 years (range 49–79 years). The mean diameters of the specimen and tumor were 34.8 mm (range 28–45 mm) and 22.7 mm (range 17–26 mm), respectively. Representative resected tissue is shown in Fig. 4a–c.

**Table 1:** Clinicopathological characteristics of the submucosal tumors

Case no.	Age (years)	Gender	Location^a^	Circumference^b^	Specimen (mm)	Tumor (mm)	Pathology
1	58	M	M	Gre	45 × 35 × 22	24 × 23 × 19	Schwannoma
2	59	M	U	Post	33 × 27 × 13	19 × 16 × 11	GIST
3	61	M	U	Post	30 × 30 × 20	26 × 26 × 17	GIST
4	71	F	U	Gre	38 × 23 × 23	25 × 23 × 23	GIST
5	79	F	U	Less	35 × 32 × 20	25 × 20 × 20	GIST
6	49	M	U	Ant	28 × 19 × 18	17 × 17 × 17	GIST

^a^The three portions of the stomach: *U* upper third, *M* middle third

^b^The four equal parts of the gastric circumference:. *Less* lesser curvature, *Gre* greater curvature, *Ant* anterior wall, *Post* posterior wall*GIST* gastrointestinal stromal tumor

Operative data for NEWS are shown in Table 2. All six lesions were successfully resected in an en bloc fashion. Intraoperative perforations occurred in two cases. The cause of the perforation was muscle injury by the endoscopic knife during mucosal cutting in case 1 and laparoscopic mucosal injury during seromuscular cutting in case 3.

**Table 2:** Operative data of our series

Case no.	En bloc resection	Perforation	Operation time (min)	Blood loss (ml)	Postoperative hospital stay (days)	Complications
1	Yes	Yes	397	30	7	None
2	Yes	(Conversion)	292	250	7	None
3	Yes	Yes	357	250	8	None
4	Yes	No	265	50	8	None
5	Yes	No	190	100	7	None
6	Yes	No	140	0	7	None

In case 2, we converted the procedure, because of poor recognition of the tumor margin, to endoscopic full-thickness resection with subsequent laparoscopic suture closure of an iatrogenic gastric defect.

After the initial three cases, we introduced the optical fiber to identify the outer portion of the tumor via endoscopy, dissected the seromuscular layer as well as the deeper layer of the submucosa laparoscopically, and doubled the amount of hyaluronate solution. In the latter three cases, the entire procedure was carried out successfully. The mean operation time and blood loss were 349 min and 177 ml in the first three cases; in the latter three cases, these were 198 min and 50 ml, respectively.

None of our cases experienced postoperative complications such as hemorrhage, anastomosis insufficiency, delayed gastric emptying, or surgical site infection. All patients started oral intake on postoperative day 2. During the mean follow-up period of 8 months (range 2–16 months), none of our patients exhibited any symptoms, and there were no changes in dietary habits.

## Discussion

We employed this new technique for six patients with gastric submucosal tumors. Although an en bloc full-thickness resection with a minimal margin was successful, we were not able to carry out this procedure without perforation in the first three patients.

One muscular perforation was observed during the endoscopic mucosubmucosal cutting in case 1. The endoscopic view was quite different from that of ESD, and the cutting line was stereoscopic and varied according to tumor size and shape. Laparoscopic cutting of the submucosa to the fullest extent possible during the laparoscopic procedure was beneficial in terms of entering the right space between the sutured muscular layer and the lifted lesion (Fig. 3b, c).

Identification of the tumor margin via pressing with the endoscopic forceps was not essential and was limited according to the tumor location. Because the endoscopic forceps moved only along the line tangential to the wall in case 2 and misalignment of the serosal marking seemed to be highly associated with pseudo-capsule injury and tumor rupture, we converted the procedure. After this case, we used the light from the fiberoptic probe of a diode laser through the gastric endoscope for guidance. The light allowed clear identification with no limitation from tumor location, and we were able to confirm the tumor margin in the latter four cases based on the illumination provided.

One mucosal micro-perforation was observed during the laparoscopic seromuscular cutting in case 3. Muscle layer thickness is known to differ according to location [15, 16]. Sufficient amounts of submucosal injections effectively prevent perforation during ESD [17]. Similarly, doubling the amount of hyaluronate solution, as well as the sequential additive injections during the procedure, were effective for avoiding mucosal tearing. As a result, we accomplished NEWS without perforations in the latter three patients after these modifications of the procedure. Continuous suturing of the seromuscular layer was safe and feasible as previously reported [13, 14].

Although this is a preliminary report and an additional larger cohort treated employing this procedure is needed to evaluate this technique before it can be considered feasible and valid, this non-opened technique for the digestive tract theoretically provides major benefits. First, postoperative inflammatory responses as well as the surgical site infection rate might show positive effects. Second, this method enables us to perform full-thickness resection while avoiding possible tumor dissemination into the peritoneal cavity, and thus it may have potential as a treatment modality even for patients with ulcerated GIST or gastric cancer with minimal risk of lymph node metastasis. Third, upper limit of tumor size safely extracted orally should be meticulously evaluated.

In conclusion, a new laparoendoscopic technique, NEWS, is one treatment option for small gastric GIST even with an ulcerated form. NEWS might have potential for resection of gastric tumors with minimal invasion.
